# Novel radionuclides for use in Nuclear Medicine in Europe: where do we stand and where do we go?

**DOI:** 10.1186/s41181-023-00211-5

**Published:** 2023-10-12

**Authors:** Maija Radzina, Laura Saule, Edgars Mamis, Ulli Koester, Thomas Elias Cocolios, Elina Pajuste, Marika Kalnina, Kristaps Palskis, Zoe Sawitzki, Zeynep Talip, Mikael Jensen, Charlotte Duchemin, Kirsten Leufgen, Thierry Stora

**Affiliations:** 1 University of Latvia, Riga, Latvia; 2 CERN, Geneva, Switzerland; 3 Riga Stradins University, Riga, Latvia; 4 Institut Laue-Langevin, Grenoble, France; 5 KU Leuven, Louvain, Belgium; 6 Riga Technical University, Riga, Latvia; 7 Paul Scherrer Institute (PSI), Villigen, Switzerland; 8 Technical University of Denmark, Kongens Lyngby, Denmark; 9 SCIPROM Sarl, Saint-Sulpice, Switzerland

**Keywords:** Radionuclides, Diagnostic, Therapy, PET/CT, PRISMAP programme

## Abstract

**Background:**

In order to support the ongoing research across Europe to facilitate access to novel radionuclides, the PRISMAP consortium (European medical radionuclides programme) was established to offer the broadest catalog of non-conventional radionuclides for medical and translational research. The aim of this article is to introduce readers with current status of novel radionuclides in Europe.

**Main body:**

A consortium questionnaire was disseminated through the PRISMAP consortium and user community, professional associations and preclinical/clinical end users in Europe and the current status of clinical end-users in nuclear medicine were identified. A total of 40 preclinical/clinical users institutions took part in the survey. Clinical end users currently use the following radionuclides in their studies: ^177^Lu, ^68^ Ga, ^111^In, ^90^Y, other alpha emitters, ^225^Ac, ^64^Cu and Terbium isotopes. Radionuclides that would be of interest for users within the next 2–5 years are ^64^Cu, Terbium radionuclide “family” and alpha emitters, such as ^225^Ac.

**Conclusions:**

Thanks to a questionnaire distributed by the PRISMAP consortium, the current status and needs of clinical end-users in nuclear medicine were identified.

## Background

Novel radionuclides for nuclear medicine can improve diagnostics of a broad spectrum of diseases. Moreover, achievements in theranostic (therapy + diagnostic) can lead to a precise and quick way from diagnosis to treatment. Theranostics is a treatment using diagnostic imaging to identify if target receptors are present on cancer cells, followed by precision radiation treatment that targets these receptors. In the last years, nuclear medicine has shown its potential in personalized medicine and targeted therapy approaches. Implementation of positron emission tomography (PET) into clinical routine and establishing novel targeted therapies in oncology have made nuclear medicine more accessible to patients (Neels et al. 2019).

Radionuclides that are used in radiopharmaceuticals have different applications: for diagnostic, therapeutic and disease monitoring purposes in nuclear medicine practice, as well as research tools (European Medicines Agency 2023). Out of the more than 3000 different radioisotopes that scientists have synthesized in the laboratory, only a handful are regularly used for medical procedures, mostly for imaging, though the interest in targeted radionuclide therapy has been growing in the last few years. One of the main limits to the development of novel radio-medicinal products is the access to radionuclides during the development and early biomedical research phases. In order to support the ongoing research across Europe to facilitate access to novel radionuclides, the PRISMAP consortium (European medical radionuclides programme) was established to offer the broadest catalog of non-conventional radionuclides for medical and translational research. One of the aims of the European medical radionuclides program is to enable this development phase by providing access to novel radioisotopes of high purity grade for medical research (PRISMAP 2021a, b).

The most frequently used PET radionuclides are the pure positron-emitters ^18^F and ^11^C, which can be produced at medical cyclotrons (Gabriela and Jacek 2012). Also, ^68^ Ga and ^111^In are used widely for positron emission tomography (PET) and single photon emission computed tomography (SPECT), respectively. ^177^Lu and ^225^Ac are used for β^−^- and α-radionuclide therapy, respectively (Müller et al. 2018).

PRISMAP “Production of high purity isotopes by mass separation” is the European medical isotope programme that federates a consortium of key European facilities such as intense neutron sources, isotope mass separation facilities and high-power accelerators and cyclotrons, with leading biomedical research institutes and hospitals active in the translation of the emerging radionuclides into medical diagnosis and treatment. PRISMAP creates a single-entry point for a fragmented user community distributed amongst universities, research centers, industry and hospitals, in a similar way as how the National Isotope Development Centre NIDC, supported by the Department of Energy (DOE), has provided radionuclide sources for users in the United States of America (USA).

PRISMAP brings together a consortium of 23 beneficiaries from 13 countries, one European research laboratory and an international organisation. It further receives support from leading associations and institutions in the field such as the European Association of Nuclear Medicine (EANM) and the International Atomic Energy Agency (IAEA) (PRISMAP 2021a, b). It supplies radionuclides across Europe and beyond to any user on an excellence basis, and offers support through a network of biomedical research facilities that are fully equipped and licensed to use the array of radionuclides available in the PRISMAP portfolio.

In order to find out the situation with novel radionuclides in Europe, the consortium questionnaire was created. The construction of a questionnaire was based on literature review, an expert review by a certain panel and finally a validating before the survey starts in consortium members meeting.

A questionnaire was disseminated through the PRISMAP consortium and user community, professional associations of radiology and nuclear medicine, and preclinical/clinical end users in Europe, collecting a total of 40 responses. The survey was based online and the first results were summarized after a 9 month long period. The total number of survey candidates is undetectable, because it was not sent out to specific institutions, but disseminated through communities and associations. Moreover, the link to this questionnaire is still available on the PRISMAP public homepage to encourage new clinical users to become visible.

Presented responses presented the current status and needs of clinical end-users in nuclear medicine. This questionnaire covered novel radioisotopes, clinical exam type and advanced technology current usage and near future plans in next 2–5 years. The survey also included questions about research and development activities and education activities that respondents’ institutions provide. Also, a literature review of currently used novel radionuclides in clinical and preclinical phases was carried out.

## Main text

### Examples of novel radionuclides usage in medicine—the review of current state in preclinical and clinical phase

**Scandium** presents three radioisotopes for theranostic application. ^43^Sc (T_1/2_ = 3.89 h, β^+^  = 88.1%, < E_β+_ >  = 476 keV) and ^44^Sc (T_1/2_ = 4.04 h, β^+^  = 94.3%, < E_β+_ >  = 632 keV) can both be used for PET, while ^47^Sc (T_1/2_ = 3.35 d, β^−^ = 100%, < E_β-_ >  = 162 keV) is the therapeutic match—also suitable for SPECT (E_γ_ = 159 keV (68.3%)). ^43^Sc and ^44^Sc can be radiolabelled with macrocyclic chelators, e.g. DOTA or other chelators such as NODAGA, AAZTA, pypa, mpatcn, etc., but ^47^Sc—with macrocyclic chelators, in particular DOTA. Currently, ^44^Sc is most advanced in terms of production, as well as with pre-clinical investigations, and has already been employed in proof-of-concept studies in patients. Even though the production of ^43^Sc may be more challenging, it would be advantageous due to the absence of high-energetic γ-ray emission (E_γ_ = 1157 keV (99.9%) for ^44^Sc). The development of ^47^Sc is still in its infancy, however, its therapeutic potential has been demonstrated preclinically (Müller et al. 2018; PRISMAP 2021a, b). The results of study showed that accurate quantitative scandium-43/44 PET/CT is achievable in commercial devices (Lima et al. 1826). **Scandium-44** was proposed as an alternative radionuclide to ^68^ Ga for PET imaging allowing the user of ^44^Sc-PSMA-617 as a diagnostic match to ^177^Lu-PSMA-617 (Umbricht et al. 2017). **Scandium-47** is a β^–^-emitter suitable for therapeutic purposes, which also produces γ-ray emission useful for SPECT imaging (Müller et al. 2018).

**Manganese-52** (T_1/2_ = 5.59 d, β^+^  = 29.4%, < E_β+_ >  = 242 keV) shows promise in positron emission tomography (PET) and in dual-modality manganese-enhanced magnetic resonance imaging (MEMRI) applications including neural tractography, stem cell tracking, and biological toxicity studies (Graves et al. 2015). ^52^Mn is suited for combined PET/MR imaging or as PET analog of Mn-based MRI contrast agents to study their in vivo distribution and pharmacokinetics (Saar et al. 2018; Lewis et al. 2015). ^52^Mn can be radiolabelled with macrocyclic chelators, such as DOTA (PRISMAP 2021a, b).

**Copper-64** (T_1/2_ = 12.7 h, β^+^  = 17.6%, < E_β+_ >  = 278 keV, β^−^ = 38.5%, < E_β-_ >  = 191 keV) labeled compounds ranging from small molecules (peptides, etc.) to antibodies are regularly used in clinics and clinical trials respectively. A recent study confirmed that radiolabeled ^64^Cu-PSMA is a promising agent to target and visualize PSMA receptor positive tumor lesions with high serum stability. This was shown in preclinical evaluation by small-animal PET studies, organ distribution and a patient application (Carlos Dos Santos et al. 2020).

**Copper-67** (T_1/2_ = 61.83 h, β^−^ = 100%, < E_β-_ >  = 145 keV) is an emerging β^−^ emitter of interest for therapy with γ-emission usable for SPECT/CT (E_γ_ = 91.3 and 93.3 keV (21.1%) and E_γ_ = 185 keV (44.2%)) that forms a theranostic pair together with ^64^Cu. Promising results in SPECT/CT imaging have been already published showing the benefit of using medium energy collimators to obtain reconstructed images of a similar quality to the ones that can be obtained using ^177^Lu (Merrick et al. 2021). ^64^Cu and ^67^Cu can be radiolabelled with macrocyclic chelators. DOTA is being used, but other, specific copper chelators such as MeCOSar may provide improved stability (PRISMAP 2021a, b).

**Palladium-103** (T_1/2_ = 17.0 d, 100% electron capture, E_X-ray_ ≈ 20 keV (64.9%) and E_X-ray_ ≈ 23 keV (12%)) is used in the development of a new concept for brachytherapy, based on gold–palladium (AuPd) alloy nanoparticles, intrinsically radiolabeled with ^103^Pd. [^103^Pd]AuPd alloy nanoparticles embedded in gel-forming liquids have been tested preclinically (Fach et al. 2021). ^103^Pd/^103m^Rh generators have been developed (Jensen et al. 2020).

**Silver-111** (T_1/2_ = 7.45 d, β^−^ = 100%, < E_β-_ >  = 350 keV) labeled hydroxyapatite particles have been studied preclinically for radiosynovectomy. Radiosynovectomy consists of intra-articular injection of a β^−^-emitting radionuclide in colloidal or particulate form, which comes into contact with synovium. The phagocytic cells absorb some of the injected dose, which is transmitted to the synovium. If the amount of radioactivity injected is large enough the tissue will be destroyed. A study has shown that regenerated tissue will be asymptomatic for 2–5 years (Chattopadhyay et al. 2008).

**Caesium-128** (T_1/2_ = 3.66 min, β^+^  = 68.8%, < E_β+_ >  = 1260 keV) is a short half-life radionuclide with high potential for application in PET. It is provided to end users as a generator ^128^Ba/^128^Cs by profiting from the decay of its longer half-life (2.5 days) parent ^128^Ba (Lagunas-Solar et al. 1982). Moreover, the radioisotope ^129^Cs (T_1/2_ = 32.06 h, E_γ_ = 372 keV (30.6%)) is also of interest for imaging due to its emissions and half-life suitable for SPECT.

**Lanthanum-135** (T_1/2_ = 18.9 h, 100% electron capture (94.6% Auger electrons), E_Auger e-_ = 2.66–5.97 keV (77.6%)) is a radiolanthanide, usually in trivalent state. ^135^La can be chelated e.g. with DTPA or DOTA (PRISMAP 2021a, b; Abel et al. 2018). It has been proposed as a therapeutic Auger electron emitter and has suitable x-rays emissions for SPECT imaging (E_X-ray_ ≈ 32 keV (61.3%) and E_X-ray_ ≈ 37 keV (13.4%)) using low energy detectors. Its similar chemistry to actinium suggests it may also be a suitable imaging partner to ^225^Ac for theranostic applications.

**Samarium-153** (T_1/2_ = 46.28 h, β^−^ = 100%, < E_β-_ >  = 225 keV) is suited as a source for Mößbauer spectroscopy, a highly sensitive technique to characterize the chemical state and chemical environment of samarium bound in solids or frozen ex vivo samples (Friedman et al. 1976). It can be radiolabelled with macrocyclic chelators, in particular DOTA (PRISMAP 2021a, b). ^153^Sm with limited specific activity is mainly used for bone pain palliation, commercially available under the brand name Quadramet®. High specific activity ^153^Sm has been recently produced using the mass separation of an activated sample, opening the door to a translation of HSA ^153^Sm for therapeutic applications (Voorde et al. 2021).

**Terbium** presents four radionuclides of medical interest: ^149^ Tb (T_1/2_ = 4.12 h, β^+^  = 7.1%, < E_β+_ >  = 730 keV, *α* = 16.7%, E_*α*_ = 3967 keV), ^152^ Tb (T_1/2_ = 17.5 h, β^+^  = 20.3%, < E_β+_ >  = 1140 keV), ^155^ Tb (T_1/2_ = 5.32 d, E_γ and X-ray_ ≈ 45 keV (107%), E_γ_ = 86.6 keV (32%), E_γ_ = 105.3 keV (25.1%)) and ^161^ Tb (T_1/2_ = 6.95 d, β^−^ = 100%, < E_β-_ >  = 225 keV, ≈ 227% conversion and Auger electrons, E_γ and X-ray_ ≈ 48 keV (43.1%), E_γ_ = 74.6 keV (10.3%)). ^149^ Tb is the only *α*-emitter with clinically useful half-life that is also suitable for PET imaging. Muller et al. presented a preclinical study with a tumor-bearing mouse. The conclusion was that the perspective of alpha-PET makes ^149^ Tb highly appealing for radiotheragnostic applications in future clinical trials (Müller et al. 2017). Similar study was conducted by Umbricht et al.—^149^ Tb-PSMA-617 was used for targeted α-therapy (TAT) using a mouse model of prostate-specific membrane antigen (PSMA)-expressing prostate cancer. The PET images confirmed the selective accumulation of ^149^ Tb-PSMA-617 in PC-3 PIP tumor xenografts. The unique characteristics of ^149^ Tb for TAT make this radionuclide of particular interest for future clinical translation, thereby, potentially enabling PET-based imaging to monitor the radioligand’s tissue distribution (Umbricht et al. 2019). A first in human PET/CT imaging was performed with ^152^ Tb-DOTATOC over 24 h (Baum et al. 2017). The results of a study by Muller et al. demonstrate the successful preparation and preclinical testing of ^152^ Tb-PSMA-617 and its first applications in a patient with metastatic castration-resistant prostate cancer (Müller et al. 2019). Terbium-155 may be of particular interest for low-dose SPECT prior to therapy with a therapeutic match such as the β^−^-emitting radiolanthanides ^177^Lu, ^161^ Tb, ^166^Ho, and the pseudo-radio lanthanide ^90^Y (Müller et al. 2014a). Due to the strong similarities of chemical and nuclear properties of ^161^ Tb and ^177^Lu, ^161^ Tb is considered a logical evolution from ^177^Lu, increasing the local dose deposition in therapeutic applications with respect to the latter. The therapeutic benefit of ^161^ Tb over ^177^Lu has been demonstrated preclinically with different compounds (Müller et al. 2014b). A clinical SPECT/CT protocol has been proposed for imaging with ^161^ Tb (Marin et al. 2020). A first-in-human application of ^161^ Tb-DOTATOC has been reported including clinical SPECT/CT imaging (Baum et al. 2021).

**Thulium-165** (T_1/2_ = 30.1 h) is a radiolanthanide, usually in trivalent state. The main application of ^165^Tm is in ^165^Tm/^165^Er generators making available non-carrier-added ^165^Er. ^165^Er (T_1/2_ = 10.3 h, ≈ 75% Auger electrons with < E_Auger_ > ≈ 5 keV, E_X-ray_ ≈ 47 keV (59.4%), E_X-ray_ ≈ 54 keV (14.3%)) is of interest for Auger therapy and has x-rays emission compatible with imaging. (Beyer et al. 2004).

**Erbium-169** (T_1/2_ = 9.39 d, β^−^ = 100%, < E_β-_ >  = 100 keV, ≈ 55% conversion and Auger electrons with < E > ≈ 7 keV) is a potential radionuclide toward therapy of metastasized cancer diseases, particularly, for the treatment of single cancer cells and small metastases. It can be produced in nuclear research reactors, irradiating isotopically-enriched ^168^Er_2_O_3_. High specific activity ^169^Er is obtained by using mass separation of the irradiated sample (Talip et al. 2021). Chelators suitable for ^177^Lu can be directly employed for ^169^Er too, in particular DOTA (PRISMAP 2021a, b).

**Gold-199** (T_1/2_ = 3.14 d, β^−^ = 100%, < E_β-_ >  = 82 keV, E_γ_ = 158.4 keV (40%), E_γ_ = 208.2 keV (8.7%)) has been used for radiolabeling and SPECT imaging of Au nanoparticles (Zhao et al. 2016). Preclinical in vivo SPECT imaging with mixed ^198^Au/^199^Au radiotracers has been reported (Fazaeli et al. 2014).

**Bismuth-213** (T_1/2_ = 45.6 min, β^−^ = 97.8%, < E_β-_ >  = 436 keV, *α*_cumulative_ = 100%, < E_*α* cumulative_ >  = 8.32 MeV) has been used preclinically and clinically with antibodies and peptides (Morgenstern et al. 2020). It can be labelled with macrocyclic chelators such as DOTA (PRISMAP 2021a, b). A clinical trial with ^213^Bi-DOTA-substance P has shown promising results for glioblastoma therapy (Krolicki et al. 2018). In spite of its short half-life, this isotope may be supplied to clinics via a ^225^Ac generator with a longer shelf life (weeks).

**Radium-223** (T_1/2_ = 11.43 d, *α*_cumulative_ = 400%, < E_*α* cumulative_ > ≈ 6.6 MeV)—free Ra^2+^ ions act analogously to Ca^2+^ as bone seekers. It is used for targeted therapy of bone metastases of metastatic prostate cancer and is the only α-emitting radionuclide currently broadly approved in clinical practice (Parker et al. 2013).

**Actinium-225** (T_1/2_ = 9.92 d, *α*_cumulative_ = 400%, < E_*α* cumulative_ >  = 6.88 MeV) is one of the most promising new radioisotopes in the fight against cancer. ^225^Ac has been used in preclinical studies for over 25 years (Beyer et al. 1997). ^225^Ac is suitable for many macrocyclic chelators used with other trivalent metallic ions, in particular DOTA (PRISMAP 2021a, b). Reports on the remarkable therapeutic efficacy of ^225^Ac-PSMA617 for therapy of prostate cancer have stimulated significant global interest in applying ^225^Ac as therapeutic nuclide in targeted alpha therapy of cancer. Moreover, further promising applications of the alpha emitters ^225^Ac include the therapy of brain tumors, bladder cancer, neuroendocrine tumors, and leukemia (Morgenstern et al. 2020). The implementation of ^225^Ac-PSMA-617 as a therapy tool for metastatic castration-resistant prostate cancer (mCRPC) lead to a major advancement in targeted alpha therapy (Fendler and Cutler 2017). It was reported that two patients with late-stage mCRPC came to complete remissions after treatment with ^225^Ac-PSMA-617 (Kratochwil et al. 2016). One more example was reported of the therapeutic efficacy of a ^225^Ac-PSMA-617 patient with mCRPC that was progressive under conventional therapy, and was treated with two cycles of ^225^Ac-PSMA-617 with a cumulative activity of 14 MBq. Restaging with ^68^ Ga- PSMA PET/CT after 5 months showed a remarkable molecular imaging response. This patient also demonstrated a biochemical response with a decrease in PSA level from 1,301 to < 0.05 ng/mL (Morgenstern et al. 2018).

### Novel radionuclides for nuclear medicine—the current perspective from clinical end users point of view in Europe

#### Respondent profile

During a PRISMAP questionnaire, a scene of nuclear medicine in Europe from clinical/preclinical end users’ point of view was identified. A total of 40 preclinical/clinical users institutions took part in the survey (55% clinical hospitals, 25% research institution-hospital collaborations, 10% preclinical research institutions, 2.5% private clinics and 7.5%—other types of institutions: 2 clinical research organisations, 1 private clinic and 1 manufacturer and ambulatory clinical nuclear medicine center).

**Fig. 1:**
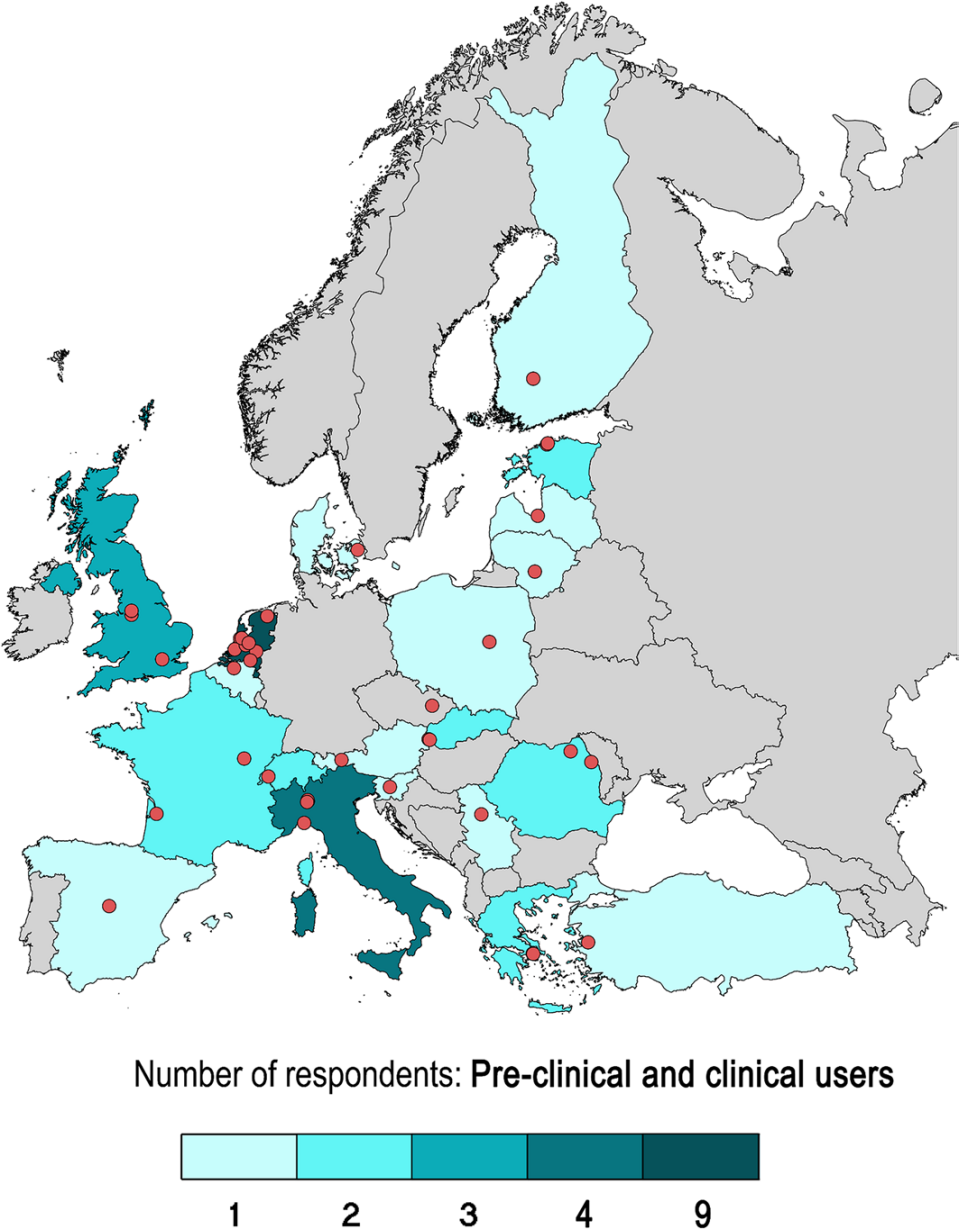
Map of respondents represented countries and cities

The respondents represented 22 countries among which 9 were coming from the Netherlands, 3 from Italy, 3 from the United Kingdom, 2 from France, 2 from Romania, 2 from Greece, 2 from Estonia, 2 from Slovakia, 2 from Switzerland, 1 each from Austria, Latvia, Belgium, Denmark, Poland, Turkey, Spain, Cyprus, Slovenia, Finland, Serbia, Czechia and Lithuania (see Fig. 1).

**Fig. 2:**
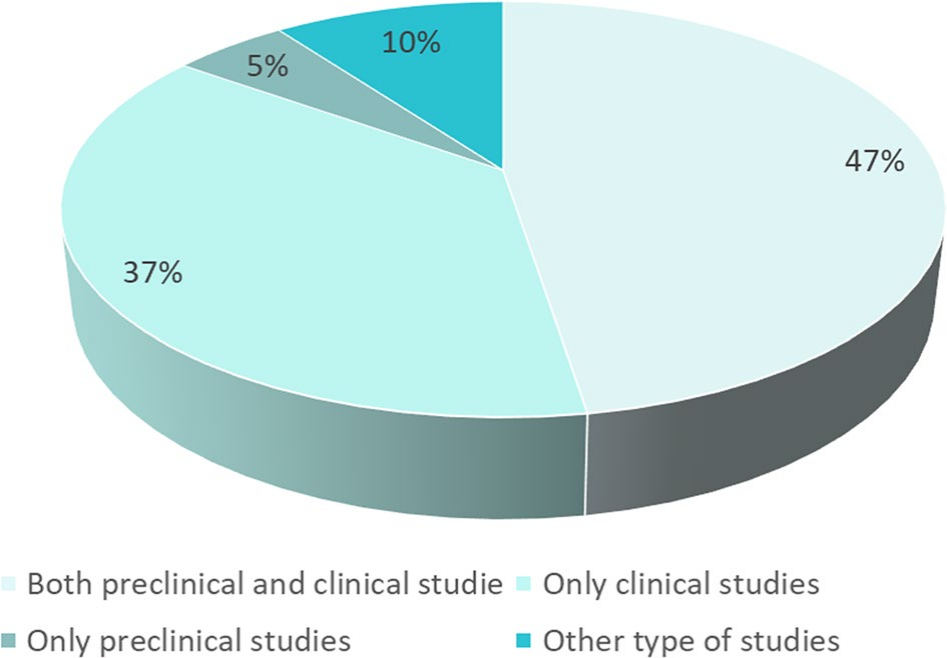
Type of respondents conducted studies

47% of the respondents conducted both preclinical and clinical studies, 37% respondents only clinical studies, 5% respondents only preclinical studies and 10% respondents other types of studies, such as medical physics, drug biodistribution, drug selection, fundamental (analytical methods), medical research. It is seen that the majority of respondents work in the clinical phase, so the results of the survey are representative of the part of the end users that use it in daily clinical practice (see Fig. 2).

**Fig. 3:**
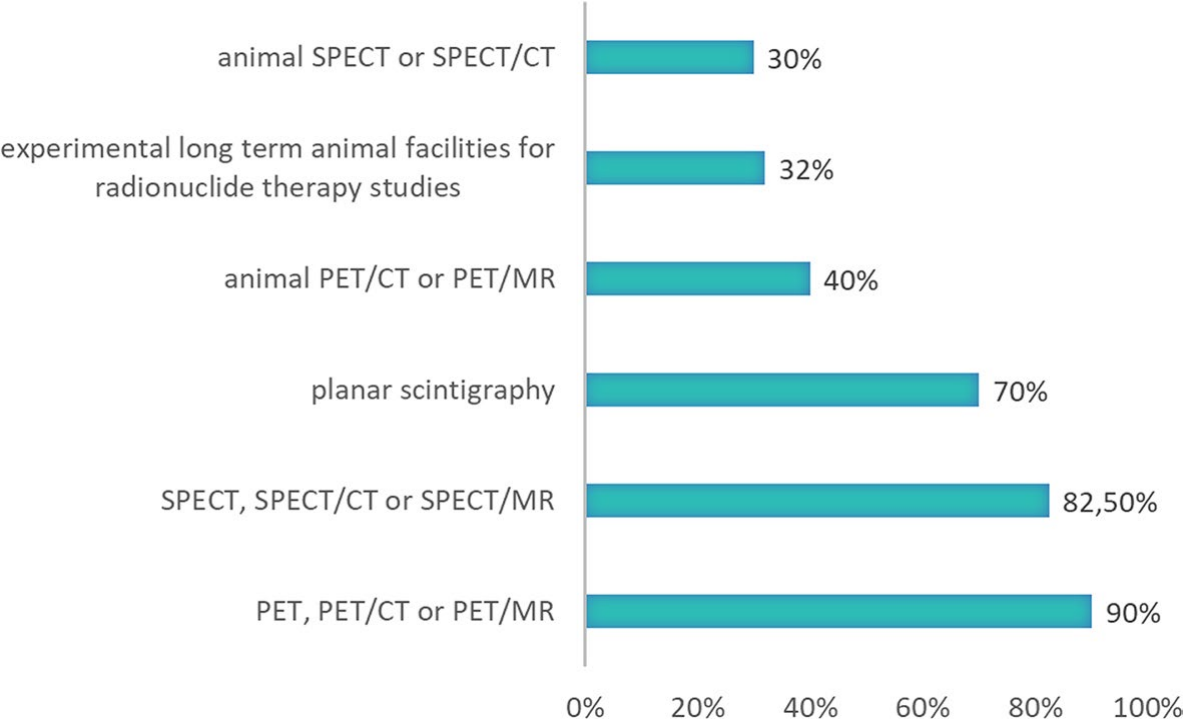
Equipment that respondent’s institutions have for daily practice

Clinical imaging is common for nearly all respondents: For routine clinical practice 90% respondents have PET, PET/CT or PET/MR, 82,5% respondents SPECT, SPECT/CT or SPECT/MR, 70% respondents planar scintigraphy, 40% respondents animal PET/CT or PET/MR, 32% respondents experimental long term animal facilities for radionuclide therapy studies and 30% respondents animal SPECT or SPECT/CT (see Fig. 3).

**Fig. 4:**
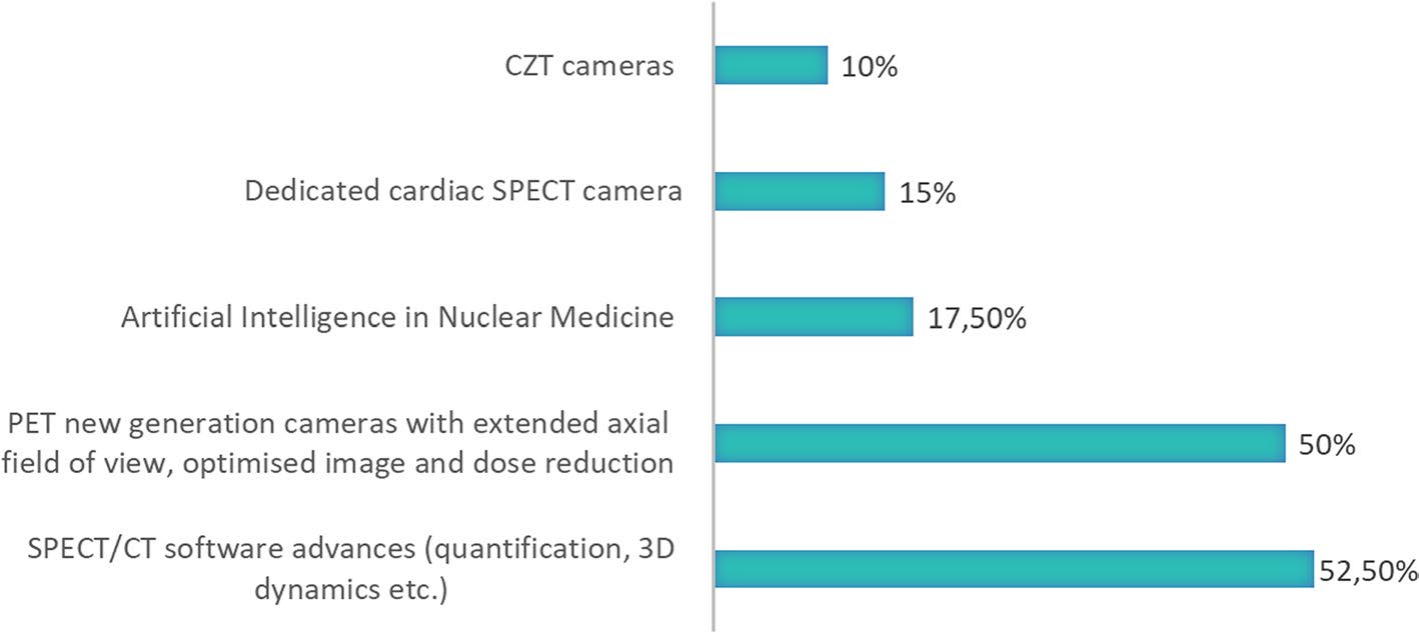
Emerging technologies that respondents' institutions use in daily work

Furthermore, many respondents are also involved in developments of those imaging protocols. The emerging technologies that the respondent’s institutions have are the following: SPECT/CT software advances (quantification, 3D dynamics etc.) (52,5% respondents), PET new generation cameras with extended axial field of view, optimised image and dose reduction (50% respondents). Respondents also mentioned the use of Artificial Intelligence in Nuclear Medicine (17,5% respondents) as well as dedicated cardiac SPECT cameras (15% respondents) and CZT cameras (10% respondents)—see Fig. 4 for more details.

**Fig. 5:**
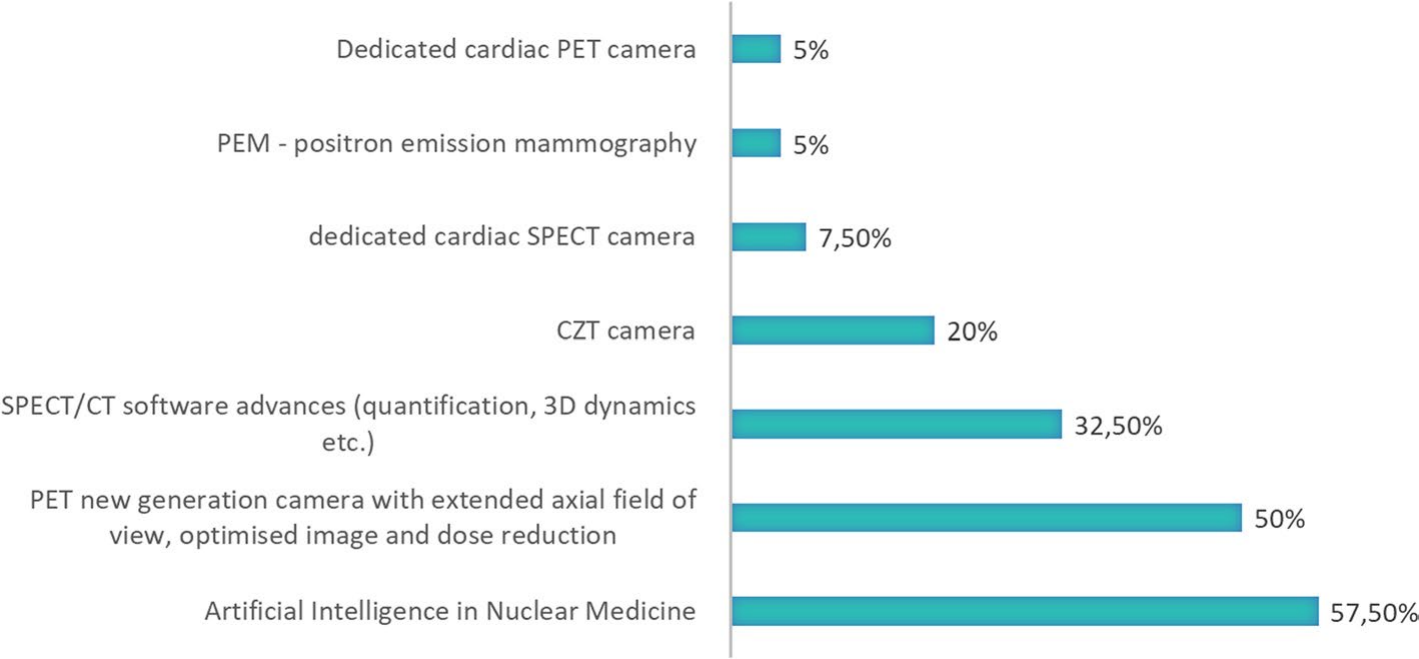
Emerging technologies that respondents want in the near future (2–5 year) in their institutions for daily work

Regarding emerging imaging technologies that respondents would like to work with in their facilities in 2–5 years, similar answers were obtained. The artificial intelligence in nuclear medicine concerned 57,5% respondents. PET new generation camera with extended axial field of view, optimised image and dose reduction was ticked by 50% respondents and SPECT/CT software advances (quantification, 3D dynamics etc.) for 32,5% of the respondents. CZT camera represented 20% respondents, dedicated cardiac SPECT camera 7,5% respondents and PEM—positron emission mammography and dedicated cardiac PET camera, 5% respondents each (see Fig. 5).

All respondents perform studies in oncology, while inflammation studies were reported from 80% respondents, cardiology—77,5% respondents, neurology—75% respondents, endocrinology—72,5% respondents, nephrology—60% respondents, pulmonology—57,5% respondents, traumatology-orthopaedics—55% respondents. A similar scene was seen about studies which respondent’s facility plan to implement within the next 2–5 years. Most abundant answers were oncology (57,5% respondents), inflammation (50% respondents), neurology and cardiology (40% respondents each), endocrinology (32,5% respondents), and pulmonology and traumatology/orthopaedics (30% respondents each).

#### Use of novel radionuclides

Part of the previous section summarized activities with radionuclides that are not only used in preclinical phase or experimental laboratories, but also used by end users in their daily clinical practice. The following “novel” non-conventional radionuclides are used by clinical users in Europe: ^177^Lu (80% respondents), ^68^ Ga (72,5% respondents), ^111^In (57,5% respondents), ^90^Y (52,5% respondents), other alpha emitters (42,5% respondents), ^225^Ac (20% respondents), ^64^Cu (15% respondents) and Terbium isotopes (10% respondents).. Other radionuclides mentioned were ^223^Ra, ^89^Zr, ^166^Ho, ^131^I, ^123^I, ^212^Pb, ^89^Sr, and ^153^Sm. Some of the radionuclides of terbium and scandium that are very promising, are not yet utilized in the clinical environment because of their poor availability at the time of the questionnaire.

**Fig. 6:**
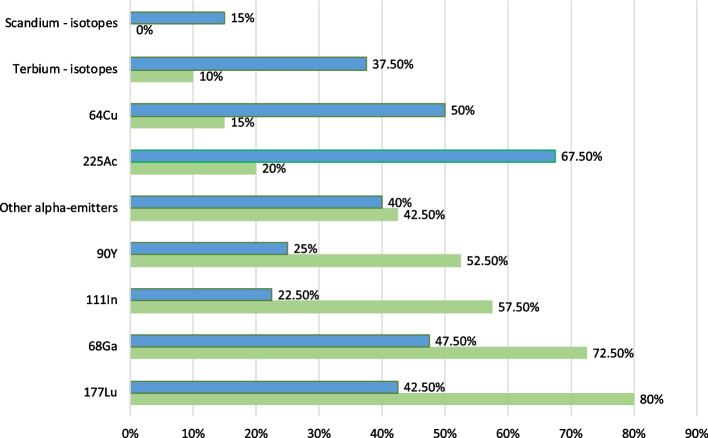
Novel radionuclides used by end users in their daily routine (green) and radionuclides that respondents would be interested to use in the next 2–5 years (blue)

Novel radionuclides that respondents would be interested to use in the next 2–5 years were ^225^Ac (67,5% respondents), ^64^Cu (50% respondents), ^68^ Ga (47,5% respondents), ^177^Lu (42,5% respondents), and other alpha emitters (40% respondents) and Terbium isotopes (37,5% respondents). Figure 6 shows both -radionuclides that end users use and radionuclides that end users would be interested in. It is seen that the demand for ^225^Ac and the terbium quadruplet (149, 152, 155, 161) coming from clinical end users will increase significantly in the following years. The foreseen application possibilities and an increasing demand for these radionuclides also point out the low availability of them at the moment, either by production capacity or required amounts or purity grade.

**Fig. 7:**
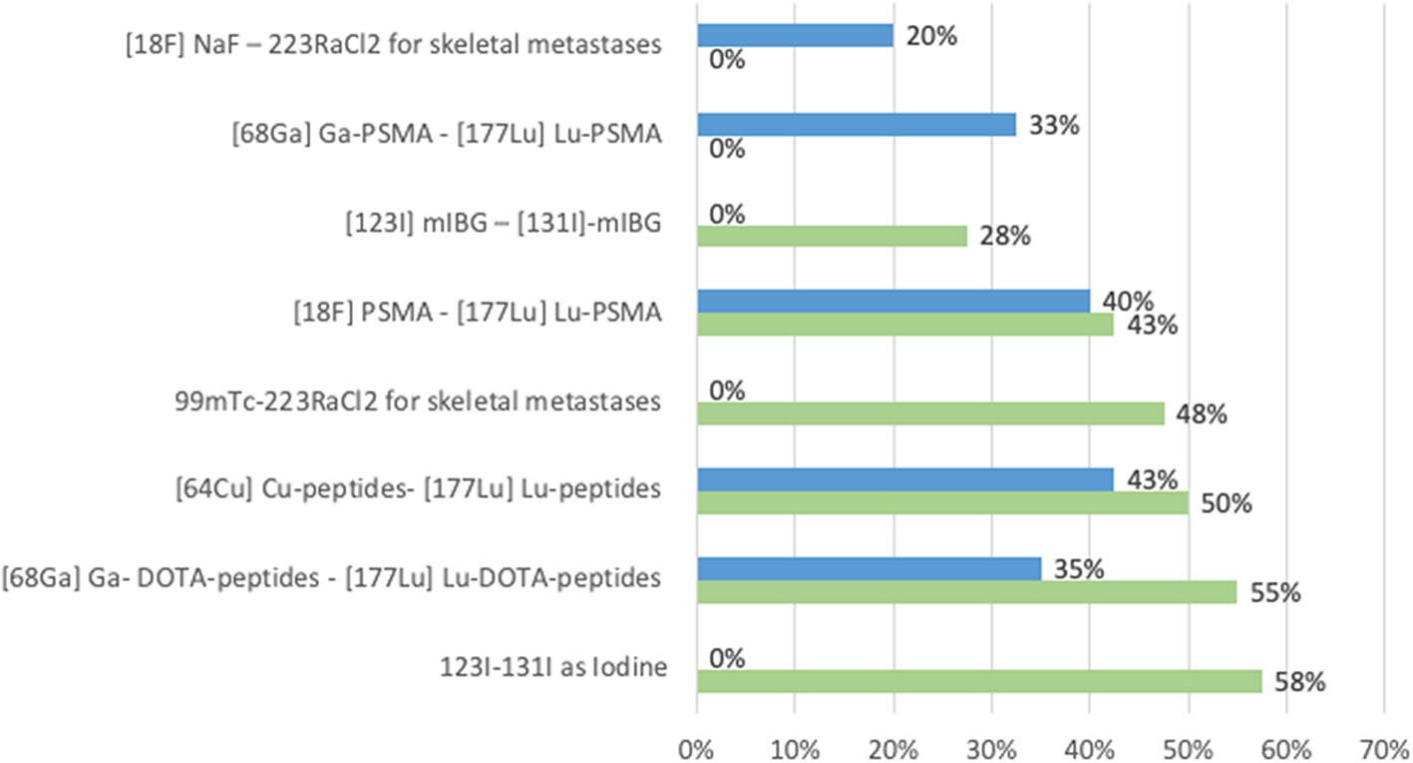
Theranostic pairs used by end users now (green) and theranostic pairs that respondents would be interested to use in the next 2–5 years (blue)

Not only single radionuclides were used in daily practice, but also theranostic pairs. If we look at the current radionuclide use for theranostics, Fig. 7 shows that the most popular pairs were ^123^I-^131^I as Iodine (57,5% respondents), [^68^ Ga] Ga-DOTA-peptides—[^177^Lu] Lu-DOTA-peptides (55% respondents), [^64^Cu] Cu-peptides—[^177^Lu] Lu-peptides (50% respondents), ^99m^Tc-^223^RaCl_2_ for skeletal metastases (47,5% respondents), [^18^F] PSMA—[^177^Lu] Lu-PSMA (42,5% respondents) and [^123^I] mIBG – [^131^I]-mIBG (27,5% respondents).

Most often mentioned theranostic pairs that respondent would be willing to use in the future were [^64^Cu] Cu-peptides-[^177^Lu] Lu-peptides (42,5% respondents), [^18^F] PSMA—[^177^Lu] Lu-PSMA (40% respondents), [^68^ Ga] Ga-DOTA-peptides—[^177^Lu] Lu-DOTA-peptides (35% respondents), [^68^ Ga] Ga-PSMA—[^177^Lu] Lu-PSMA (32,5% respondents) and [^18^F] NaF—^223^RaCl_2_ for skeletal metastases (20% respondents), as presented in Fig. 7.

We observed that none (except for iodine radiopharmaceuticals) of the current responder institutions yet are interested in possibilities of “matched pair” from terbium and scandium radionuclides in the near future. This probably reflects the still insufficient pre-clinical data and/or availability of such radionuclides.

#### Improvements for daily practice

The following improvements were mentioned by preclinical/clinical users as their need for daily practice: unified licensing and registration of available radionuclides and kits in Europe (80% respondents), information about transport and logistics network in Europe (55% respondents), database of available radionuclides and the geographic location of the supply site (50% respondents), and some specified equipment/technologies (e.g., collimators etc.) (45% respondents). On-site training with the visit of international experts is also a wish (40% respondents) as is outsourced crucial training for technical personnel (32,5% respondents) and medical doctors (30% respondents).

Some of the respondents' countries send patients to other countries for specified nuclear medicine examinations and/or treatment procedures. The reason of this outsourcing is due to unavailability of the specific radiopharmaceuticals (42,5% respondents), a lack of reimbursement by the national healthcare system (22,5% respondents), and unavailable radionuclide for radiopharmaceutical production (17,5% respondents).

#### Research and development activities

Out of all respondents from preclinical/clinical institutions, 87,5% mentioned that their research and development activities would benefit from collaboration/cooperation in obtaining emerging radionuclides with centralized and harmonized procedures and legislation, offered by efforts of the PRISMAP consortium.

One of the main interest of end users were novel radionuclides—some respondents indicated specific ones such as ^43^Sc/^44^Sc and ^64^Cu/^61^Cu and some indicated novel radionuclides overall—clinical translation of novel theranostic tracers, higher availability of new radiopharmaceuticals, access to new radionuclides for novel radiopharmaceuticals, enhancing clarity and regulatory procedures to enhance research with radiopharmaceuticals, improving the delivered radionuclide data and regulation, along with biomedical research capacity and speed up implementation of new radionuclides and/or broader availability of currently used radionuclides. Production of novel theranostics pairs and access to rare and exotic radionuclides and need for quick registration of radiopharmaceuticals were also mentioned.

Respondents indicated the interest in collaborating with PRISMAP consortium members with regards to the sharing of new research protocols across countries, speeding up the implementation of new radiotracers in clinical practice, extending of portfolio in performing preclinical studies. Also interest in multicentric clinical trials, international studies, and research and clinical use interest were present.

Finally, technical interests from end users were indicated, including the standardization and harmonization of the medical physics calibrations, interests to establish labeling of different probes (mostly peptide based) with different emerging radionuclides in order to promote translation to the clinic.

#### Education

77,5% of all end user institutions are involved in the training of industry experts, technicians, students, and researchers at various expertise levels.

**Fig. 8:**
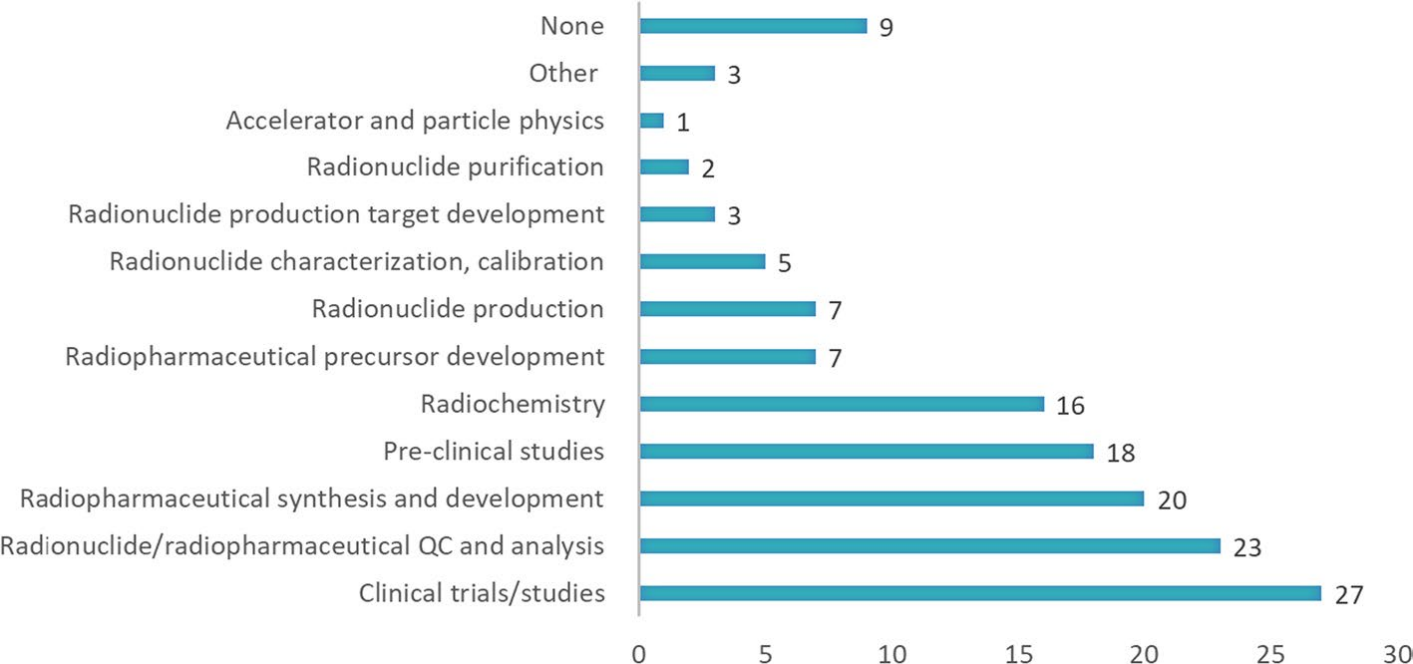
Training and knowledge transfer fields provided by respondent’s institutions

The most popular training fields are clinical trials/studies (27 respondents), radionuclide/radiopharmaceutical QC and analysis (23 respondents), radiopharmaceutical synthesis and development (20 respondents), pre-clinical studies (18 respondents), radiochemistry (16 respondents). See Fig. 8 for provided training fields from end users institutions.

**Fig. 9:**
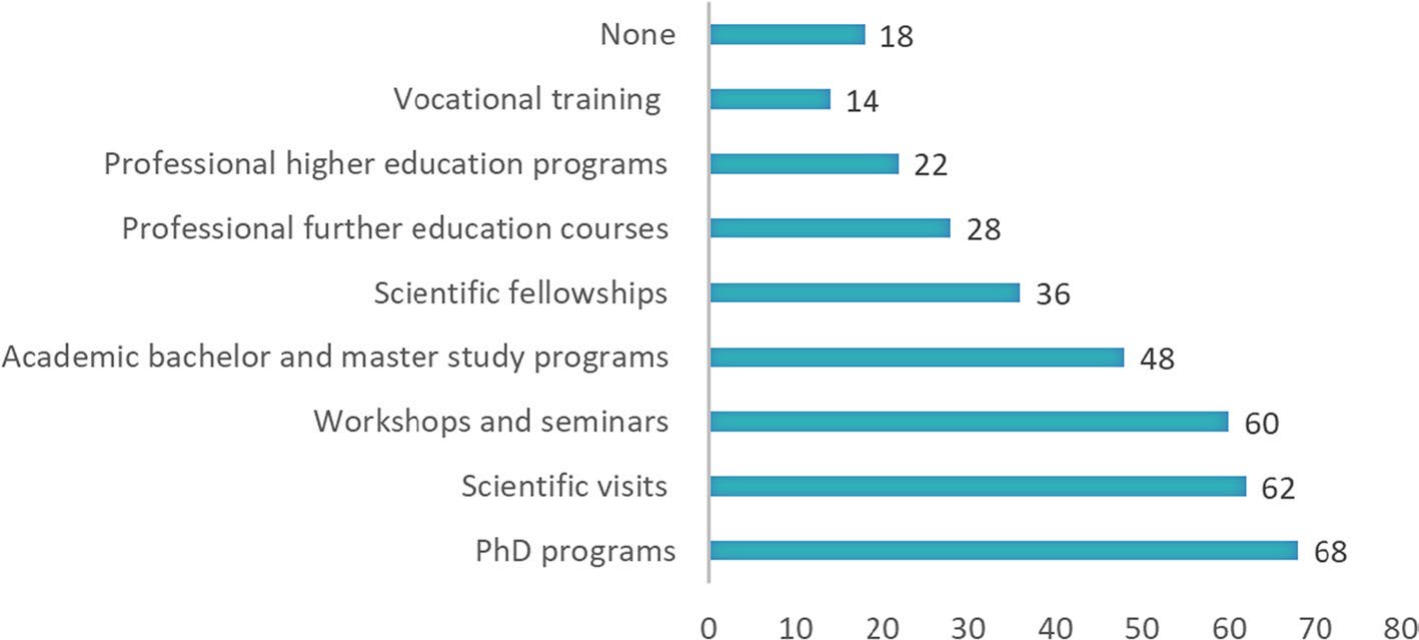
Types of provided training in nuclear physics, radiochemistry and radiopharmacy

The most often mentioned answers about training level were workshops and seminars (26 responses), PhD programmes (21 respondents), and scientific visits (21 respondents). See Fig. 9 for other types of provided training.

The target audience for training provided at respondent institutions are students (31 respondents), early-stage researchers (28 respondents), technologists (27 respondents), experienced researchers (17 respondents), nurses (14 respondents), and industry professionals (5 respondents).

The main limitations in the training process were identified as a lack of integration of radiopharmaceutical research in faculty courses and student curriculum, no dedicated program for nuclear medicine physicians, technologists and radiochemists in university, limited training capacity to 1–2 weeks, due to daily workflow, and limited time resource overall.

Respondents also mention a lack of personnel since there is a lack of trained preclinical scientists with a global view from radiochemistry to pharmacology and limited number of trainers overall. Some respondents indicated problems with training process regulation: training depends on the national regulatory organizations or there is a need to work within national health service training schemes.

It has also been indicated that there is insufficient access to radioisotopes, also a lack of equipment such as small animal imaging facilities and emerging new technologies. There is also a need for a good overview/database of training possibilities. Some respondents indicated that in their countries they don`t have any professional training available for radiopharmacy or radiochemistry, so their radiopharmacists are trained abroad**.**

## Discussion

The most frequently used radionuclides in radiopharmaceuticals that are used in daily clinical practice are Carbon-11, Fluorine-18, Nitrogen-13, Oxygen-15, Copper-64, Zirconium-89, Iodine-124, Gallium-68, Iodine-13, Technetium-99 m, Indium-111, Lutetium-177, Yttrium-90, Iodine-131, Actinium-225, Astatine-211, Radium-223 and Bismuth-213 (Malcolm et al. 2019; Al-Toubah and Strosberg 2018).

These radionuclides represent only a part of overall potential as many novel radionuclides have entered the preclinical phase studies. Due to that, the European medical radionuclides programme PRISMAP was established to offer the broadest catalog of non-conventional radionuclides for medical and translational research (PRISMAP 2021a, b).

Thanks to a questionnaire distributed by the PRISMAP consortium, the current status and needs of clinical end-users in nuclear medicine were identified. It was found out that novel radionuclides in which respondents would be interested in the near future were ^225^Ac, ^64^Cu, ^68^ Ga, ^177^Lu, other alpha emitters and Terbium isotopes. A lack of training possibilities and trained personnel have been identified as well as access to radionuclide to gain experience.

Also, the study has some limitations. First of all, it needs to be addressed that the study was conducted in a relatively small end users’ cohort. Not all European countries were covered—the majority of responses come from Western Europe, most notably the Benelux, France and Italy. More emphasis will be needed for reaching out to responders from South-East Europe regions. Secondly, the PRISMAP programme continues and these results are preliminary, limited outcomes of this study so far. Moreover, study should be extended outside the PRISMAP consortium with the possibility to obtain data from other preclinical/clinical end users in Europe.

## Conclusions

The current perspective shows that nuclear medicine specialists/clinical end users from broad parts of Europe are interested not only in new radionuclides for diagnostics, but also in therapy and technology advancements that confirm their interest in development. This study was preliminary and should be extended outside the PRISMAP consortium.

## Data Availability

The datasets generated during the current study are available from the corresponding author on reasonable request.

## Abbreviations

PET: Positron emission tomography
SPECT: Single photon emission computed tomography
NIDC: National isotope development centre
DOE: Department of energy
EANM: European association of nuclear medicine
IAEA: International atomic energy agency
MEMRI: Manganese-enhanced magnetic resonance imaging
PET/CT: Positron emission tomography-computed tomography
SPECT/CT: Single photon emission computed tomography-computed tomography
HSA: High specific activity
TAT: Targeted α-therapy
PSMA: Prostate-specific membrane antigen
mCRPC: Metastatic castration-resistant prostate cancer
T_1/2_: Half-life
d: Day
h: Hour
β^**+**^: Positron from β^+^ decay
β^**−**^: Electron from β^−^ decay
γ: Gamma quantum
keV: Kilo-electronvolt
E: Energy
